# Description and Codification of *Miscanthus* × *giganteus* Growth Stages for Phenological Assessment

**DOI:** 10.3389/fpls.2017.01726

**Published:** 2017-10-09

**Authors:** Mauricio D. Tejera, Emily A. Heaton

**Affiliations:** Department of Agronomy, Iowa State University, Ames, IA, United States

**Keywords:** BBCH, morphology, phenology, phenophase, phyllochron, perennial C4 grass, bioenergy, senescence

## Abstract

Triploid *Miscanthus* × *giganteus* (Greef et Deu. ex Hodkinson et Renvoize) is a sterile, perennial grass used for biomass production in temperate environments. While *M*. × *giganteus* has been intensively researched, a scale standardizing description of *M*. × *giganteus* morphological stages has not been developed. Here we provide such a scale by adapting the widely-used Biologische Bundesanstalt, Bundessortenamt, CHemische Industrie (BBCH) scale and its corresponding numerical code to describe stages of morphological development in *M*. × *giganteus* using observations of the “Freedom” and “Illinois” clone in Iowa, USA. Descriptive keys with images are also presented. Because *M*. × *giganteus* plants overlap in the field, the scale was first applied to individual stems and then scaled up to assess plants or communities. Of the 10 principal growth stages in the BBCH system, eight were observed in *M*. × *giganteus*. Each principal stage was subdivided into secondary stages to enable a detailed description of developmental progression. While *M*. × *giganteus* does not have seed development stages, descriptions of those stages are provided to extend the scale to other *Miscanthus* genotypes. We present methods to use morphological development data to assess phenology by calculating the onset, duration, and abundance of each developmental stage. This scale has potential to harmonize previously described study-specific scales and standardize results across studies. Use of the precise staging presented here should more tightly constrain estimates of developmental parameters in crop models and increase the efficacy of timing-sensitive crop management practices like pest control and harvest.

## Introduction

*Miscanthus* × *giganteus* is an interspecific hybrid of *M. sacchariflorus* and *M. sinensis* (Greef et al., 1997; Hodkinson and Renvoize, 2001) with C_4_ photosynthesis and a perennial growth habit. It is native to East Asia but sterile triploid clones are now used as biomass crops in temperate environments around the globe, where they are typically characterized by relatively high biomass yields, moderate cold tolerance and low input requirements (Clifton-Brown et al., 2007; Christian et al., 2008; Heaton et al., 2008; Arnoult and Brancourt-Hulmel, 2015). Triploid *M*. × *giganteus* has these characteristics in part because it produces an annual crop of harvestable stems from a perennial root/rhizome complex that enables efficient inter-annual nutrient cycling (Cadoux et al., 2012; Dohleman et al., 2012).

A major target of perennial grass improvement programs is to develop genotypes with variant plant morphology and phenology that allow more efficient resource capture or improved feedstock quality (Jones et al., 2015). To advance understanding of phenology in *Miscanthus* and related species, we propose using a common morphological development scale based on triploid *M*. × *giganteus*, since it is both a commercial crop and a frequently used control species. Adopting common, numerical naming conventions for morphological stages would allow unambiguous and quantitative characterization of *M*. × *giganteus* development, as well as its seasonal timing i.e., phenology (Sanderson et al., 1997), as has been done in other major crops. Clearly describing when a plant goes through a particular developmental stage is beneficial because it lets researchers track and compare factors influencing that timing, such as, temperature, rainfall, photoperiod, genetics, or stress. Morphological development descriptions are also crucial to practitioners who need to manage the crop at physiologically important times (e.g., for pest control and harvest) that are better described by a development stage than a substitute metric like Julian day or thermal time.

There are several commonly used morphological development scales able to characterize plant principal growth stages but they differ in the precision with which they describe intermediate growth stages. For example, the widely used scales of Feekes (Feekes, 1941; Large, 1954) and Haun (1973) could be used to assess *Miscanthus* spp., but only in a limited way. Because these scales were developed for cereals they lack detail needed to describe the entire growth cycle of perennial grasses, and have coarse resolution in the vegetative stages (see Landes and Porter, 1989 for further comparison between scales). By contrast, Moore et al. (1991) developed a scale for forage grasses that emphasizes vegetative stages, but does not provide fine resolution of reproductive stages, limiting the scale's utility for some traits of interest, such as, flowering time and seed development.

To date, the *Miscanthus* community has opted to develop entirely new morphological development scales to meet particular research needs (e.g., Hastings et al., 2009; Miguez et al., 2009; Jensen et al., 2011; Robson et al., 2012; Zub et al., 2012; da Costa et al., 2014; Purdy et al., 2015; Trybula et al., 2015). These scales function well in a specific study or research area, but do not translate well for broader use. For example, none of them provide a complete description of all plant developmental stages, nor enough detail within each stage to accurately track development through a growing season. Therefore, *Miscanthus* spp. morphological development descriptions extant in the literature today represent stand-alone descriptions which are difficult to apply more broadly.

The Biologische Bundesantalt, Bundessortenamt and CHemische Industrie (BBCH) is another widely used scale that has been adapted for more than 50 species (Meier et al., 2009; Martínez-Nicolás et al., 2016) including the commercially used C_4_ grasses sugarcane (*Saccharum officinarum* L.; Bonnett, 2013), switchgrass (*Panicum virgatum* L.; Sanderson et al., 1997), sweet sorghum (*Sorghum bicolor* L. Moench; Dalla Marta et al., 2014), and maize (*Zea mays* L.; Lancashire et al., 1991). The BBCH system has been adopted around the globe because it has a flexible but consistent framework that facilitates comparison across diverse plant types, an especially useful feature for assessing bioenergy genera with very different features, e.g., *Cynara* (Archontoulis et al., 2010) and *Miscanthus*.

The BBCH framework consists of a two digit decimal code based on Zadoks et al. (1974) decimal code for cereals (Lancashire et al., 1991). The first digit corresponds to a *principal growth stage* (0–9) and subdivides the developmental cycle of the plant into 10 clearly recognizable and distinguishable stages (Hess et al., 1997). The second digit corresponds to a *secondary growth stage* (0–9) and describes the intermediate stages within a principal growth stage and progression through those stages. Depending on the principal stage, secondary stages correspond to either ordinal or percentage values. The two digit code consists of a combination of the principal growth stage code (tens place) and secondary stage code (ones place). In cases where further precision is needed, secondary stages can be subdivided by incorporating mesostages and extending the code to a three digit code. Characterizing morphological development of *Miscanthus* spp. with the BBCH scale would allow comparison of *Miscanthus* spp. development with that of other species for which the BBCH scale has been adapted and provide clear developmental benchmarks by which to assess and compare phenology.

The purpose of this paper is to modify the BBCH scale to describe the morphological development of *M*. × *giganteus*. Here, we (1) modify the BBCH scale to describe and codify *M*. × *giganteus* growth stages; and (2) describe and demonstrate use of the modified BBCH scale to assess phenology, specifically the onset, duration and abundance of phenological events. We hope this scale will be used by the *Miscanthus* research community to characterize development and phenology, and facilitate transparency and comparison between studies. We further hope this scale will assist management decisions for *M*. × *giganteus* producers.

## Materials and methods

To modify the BBCH scale for *M*. × *giganteus* we first developed a draft scale from similar scales in the literature. We then refined that draft scale to a robust, functioning scale using targeted *M*. × *giganteus* field observations from 2 years and three locations in Iowa, USA. We next demonstrated how the scale can be used to perform phenology assessments. We did that by adapting published practices to collect morphological data, spatially upscale phenological stages, and model developmental progression to assess phenology. This allows calculation of the date at which a given stage was reached, i.e., the onset date (Cornelius et al., 2011), and measures of developmental progression. i.e., stage duration and abundance. To clarify these procedures, we present an explanatory dataset containing biweekly measurements of *M*. × *giganteus* development over one growing season. This explanatory dataset is not meant to provide a full phenological assessment of *M*. × *giganteus*, but instead demonstrate the procedures needed to do such assessments.

### Modifying the BBCH scale to *M. × giganteus*

First, we developed a draft *M*. × *giganteus* scale using published descriptions for the closely related species sugarcane (Bonnett, 2013) and switchgrass (Sanderson et al., 1997). Typically the BBCH scale only considers development of the main stem (Meier et al., 2009), but *M*. × *giganteus* does not have a clear main stem, nor is it easy to distinguish individual *M*. × *giganteus* plants in the field. Therefore, terminology and images were adjusted to consider development of individual stems.

Second, we adjusted the draft scale to allow for both sterile and fertile *Miscanthus* genotypes. Since *Miscanthus* crops are dominantly rhizomatous, seed germination stages were replaced by rhizome growth and emergence stages. Similarly, while today's commercially available *M*. × *giganteus* clones are sterile triploids that do not sexually reproduce, the scale has stages related to seed development, enabling assessment of seeded genotypes (Clifton-Brown et al., 2017). Grain filling stages (e.g., milk and dough) were described based on generic grass descriptions (Meier, 2001) as modified for perennial grasses (i.e., *P. virgatum*) by Sanderson et al. (1997) (see Tables 1, **3**).

**Table 1:** Overview of locations and plant material used to refine *Miscanthus* × *giganteus* morphological development descriptions for the BBCH scale.

**Sites in Iowa, USA**	**Location (lat., long.)**	**Clone**	**Planting year**	**Soil class**	**Mean air temperature (°C)**	**Precipitation (mm)**
Allee farm	42.586, −95.012	Freedom	2015, 2016	Typic endoaquoll	10.0 ± 11.4	908.2 ± 19.6
Sorenson farm	42.013, −93.744	Freedom Illinois	2015, 2016 2009	Typic endoaquoll	11.1 ± 11.3	940.8 ± 14.7
South East Research Farm	41.201, −91.488	Freedom	2015, 2016	Aquic argiudoll	12.0 ± 11.1	865.6 ± 15.7

*Annual temperature and annual precipitation were averaged over observation years (2015–2016)*.

Third, we presented mesostages as tenths of the secondary stage instead of extending the code to a whole-number three digit code. This facilitates arithmetic operations and comparison within and between studies by keeping values between 0 and 99.

### Refining the BBCH scale with *M. × giganteus* field observations

To refine the literature-based draft scale for real-world application, we supplemented it with information from field observations. Observations were made of *M*. × *giganteus* clone “Freedom” grown in three locations across Iowa, USA and clone “Illinois” grown only at the Sorenson farm in central Iowa, over two growing seasons (Table 1). These observations spanned two degrees of latitude, three degrees of longitude, two plant ages (juvenile and mature), and a range of soil, climate, and fertility conditions. At each of the three locations, rhizomes of *M*. × *giganteus* clone “Freedom” (sourced from Repreve Renewables, now AgGrow Tech, Greensboro, NC, USA) were planted in both 2015 and 2016 as part of a larger study that included five nitrogen fertilization rates (0, 112, 224, 336, and 448 kg ha^−1^). Additionally, at the Sorenson farm, rhizomes of *M*. × *giganteus* clone “Illinois” (sourced from Caveny Farm, Monticello, IL, USA) had been planted in 2009. All stands were planted with 0.6 m spacing between rows and approximately 0.4 m spacing between plants within the row. During the 2015 and 2016 growing seasons, stands at the Sorenson farm (with both clones and stand ages) were used to gather observations, descriptions, images, and explanatory data. The other two locations were then used to identify gaps in the morphological description and ensure broad transferability of the scale. Stands were healthy without disease or stress symptoms during the period of study.

Morphological development information used to characterize, supplement, and refine descriptions of *M*. × *giganteus* developmental stages was collected biweekly at the Sorenson farm beginning in early spring at planting/emergence and continued until mid-winter when stems had senesced. While morphological descriptors of aboveground biomass were developed using both clones, rhizome bud development was assessed using only “Freedom” rhizomes. These stages describe morphological growth of two structures (i.e., bud swelling, first lamina expansion), and no discernible differences from the “Illinois” clone were observed based on our previous work with that clone (Heaton et al., 2008; Boersma and Heaton, 2014). To facilitate observations, rhizomes used for rhizome bud development characterization were kept unplanted under temperature and moisture conditions favorable for growth (~25°C and <2 kPa water vapor pressure deficit).

At each biweekly observation, a random sample of stems was collected from the observation stands and described according to the draft scale. The draft scale was then iteratively adjusted as needed to match field observations, and supporting images taken. In total, the complete morphological characterization was based on at least 200 randomly selected stems per morphological development stage. Descriptions of principal and secondary growth stages were organized to match existing BBCH scale descriptions, creating a working *M*. × *giganteus* scale.

### Assessing phenology using modified BBCH scale

To demonstrate assessment of *M*. × *giganteus* phenology, we collected a time series of morphological development data additional to those used in developing the *M*. × *giganteus* BBCH scale (section Refining the BBCH Scale with *M*. × *giganteus* Field Observations). We compiled these explanatory data by recording all the morphological development stages present in a random sample of ten stems of 2-year old *M*. × *giganteus* at Sorenson farm biweekly from late spring to late fall 2016. We adapted the following methodological guidelines presented by the USA National Phenology Network (Denny et al., 2014) to standardize phenological data collection.

1. *Make repeated observations of the stage status*. This provides temporal information on the presence/absence and duration of the developmental stage.
2. *Make multiple observations per location*. Multiple random stems should be observed on each date. This helps quantify temporal and spatial variation and allows scaling to larger areas like a field or population. Additionally, it gives information about stage abundance and commonality, allowing better characterization of phenological patterns. Repeated observations in space and time also enable identification of cohort emergence across the growing season. Sampling can be targeted to the cohort of interest, or used to characterize variability across the population.
3. *Independently track multiple stages occurring in parallel*. This is of special interest since morphological stages occur at the same time during the *Miscanthus* growing cycle, e.g., leaf growth and stem elongation. Typically users of morphological development scales stop tracking one stage once the next begins, but users should be able to measure progression through parallel stages if desired.

#### Estimating growth stage progression

We next used our morphological development time series data to demonstrate how growth stage progression can be determined using two metrics: cumulative abundance of a stage and development rates between stages. For the former, we followed and recommend the method developed by Schirone et al. (1991) to estimate onset duration and abundance of morphological growth stages. Cumulative abundance is calculated as the number of stems that have passed a defined developmental threshold, and presents abundance as a proportion of the total stems measured. At each sampling date, the cumulative abundance of a considered stage is estimated as the number of stems at the threshold stage or higher, divided by the sample size. When graphed, this temporal series describes a sigmoid curve, which can be modeled with a logistic function. The onset date is estimated by modeling a curve through the measured data points, then identifying the point in time at which the frequency of individuals at the threshold stage equals 50%. Onset dates for each secondary growth stage should be calculated separately within each principal growth stage to elucidate overlapping *M*. × *giganteus* stages.

Principal growth stage development rate (PGSDR) was calculated as the rate of appearance of new secondary stages over time. This rate specifies the time required to develop morphologic structures (e.g., leaves, nodes). If the relationship of development to time is linear, PGSDR is the slope of the linear regression between secondary growth stage progression and time. In case of a non-constant rate during the growing season, multiple linear regressions for multiple constant rates or non-linear models can be used. In the latter case, the derivate of the function may be more informative.

In principal growth stage 1, PGSDR corresponds to leaf appearance rate and its inverse is equivalent to the phyllochron, or accumulated time (in days or thermal time) required for the appearance of successive leaves on a stem (Xue et al., 2004). Leaf appearance rate and phyllochron have direct application to crop modeling, crop management, assessment of abiotic stress, and cultivar selection.

Leaf duration, defined as the time between leaf emergence and senescence, is another important parameter in plant phenology, crop management and modeling. When principal growth stage senescence is described in terms of number of senesced leaves and secondary growth stages attributed in the same mode as in leaf development (one every two senesced leaves), leaf duration can be estimated as the difference in onset dates for same secondary growth stage between principal growth stage 1 and 9.

We used our explanatory data set to demonstrate calculation of leaf appearance rate and phyllochron as a function of both calendar days and thermal time. Thermal time was measured in growing degree days (GDD, °C day) as:

$$\begin{array}{l} GDD=\;\left[\frac{T_{\text{max}}-\;T_{\text{min}}}{2}\right]-\;T_{b} \end{array}$$

Where *T*_max_ and *T*_min_ are daily maximum and minimum air temperature, respectively, and *T*_*b*_ is the base temperature below which development does not occur. We used 6°C as the base temperature for leaf expansion (Farrell et al., 2006) and considered [(*T*_max_ − *T*_min_)/2] = *T*_*b*_ when [(*T*_max_ − *T*_min_)/2] < 0 [see McMaster and Wilhelm, 1997, for further detail on the importance of clarifying how to deal with [(*T*_max_ − *T*_min_)/2] < 0]. Cumulative degree days were calculated as the sum of daily GDD across the growing season.

For a complete and concise description of principal growth stage progression, the cumulative thermal time should be reported for each principal stage. This could be calculated as the difference between first and last secondary growth stage onset dates. Also, using the development rate approach, it can be estimated as the difference between time at the last secondary growth stage and the first one. This approach has the advantage that it allows estimation of the beginning of the principal growth stage as the root of the describing polynomial.

#### Calculating growth stage and summary statistics

Summary statistics allow the scaling of stem-based observations to the field or population level. The method presented here has been modified from Kalu and Fick (1981) and Moore et al. (1991). Since *M*. × *giganteus* may have multiple principal growth stages running in parallel, summary statistics can be calculated separately for each stage (Equations 1, 2). Based on a sample of *N* stems, the average morphological stage at growth stage *i* (${\bar{BBCH}}_{i}$) is calculated as:

(1) $$\begin{array}{l} {\bar{BBCH}}_{i}=\frac{\sum_{j\;=\;1}^{N}BBCH_{ij}}{N} \end{array}$$

Where *BBCH*_*ij*_ represents the BBCH code for the *j*th stem in the *i*th principal growth stage. Essentially, ${\bar{BBCH}}_{i}$ estimates the average BBCH code for each individual principal stage. The complete developmental stage (*BBCH*_*complete*_) would be presented as all the average codes for each stage separated by oblique strokes. Alternatively, if users stop tracking one stage once the next begins, the complete code is the two digit code associated with most developed stage.

The variability across samples is determined for each principal growth stage individually as the standard deviation for principal growth stage *i* (*S_BBCH_i__*), calculated as:

(2) $$\begin{array}{l} {S_{BBCH}}_{i}=\;\sqrt{\frac{\sum_{j=1}^{N}{\left({\bar{BBCH}}_{i}\;-\;BBCH_{ij}\right)}^{2}}{N}} \end{array}$$

*S_BBCH_i__* is useful to estimate the variability that exists within an *M*. × *giganteus* stand at each principal growth stage and help to compare the progression of each stage. A small *S_BBCH_i__* indicates the majority of stems are at similar level of progression within a given principal growth stage, while a large *S_BBCH_i__* indicates that there is a wide range of maturity within a growth stage.

## Results

We modified the BBCH scale to describe morphological development stages of *M*. × *giganteus* using peer-reviewed literature supplemented by field observations made over a range of plant age and growth conditions. Below we present descriptions of *M*. × *giganteus* principal growth stages (Table 2) and use morphological development data to demonstrate the calculation of stage summary statistics as well stage onset, duration, and abundance for phenology assessment.

**Table 2:** *Miscanthus* × *giganteus* morphological development stages according to the BBCH scale.

**Code**	**Description**
**PRINCIPAL GROWTH STAGE 0: BUD DEVELOPMENT**	
00	Dormant rhizome
01	Beginning of bud swelling
03	End of bud swelling
05	Bud breaking: rolled leaves growing towards the surface
07	Elongation of chlorotic laminae
09	Emergence of rolled leaves through soil surface
**PRINCIPAL GROWTH STAGE 1: LEAF DEVELOPMENT**	
10	First visible leaf laminae
11	2 Fully expanded leaves
12	4 Fully expanded leaves
13	6 Fully expanded leaves continues until
…continues until…	
19	18+ Fully expanded leaves
**PRINCIPAL GROWTH STAGE 2: TILLERING**^‡^	
20	No tillers on main shoot
20.1	Partially swollen axillary bud (~2mm)
20.5	Swollen axillary bud (~8-1.0mm)
20.9	Bud breaking
21	1 Tiller on main shoot
22	2 Tillers on main shoot
…continues until…	
29	9 Tillers on main shoot
**PRINCIPAL GROWTH STAGE 3: STEM ELONGATION**	
30	Pseudo stem elongation
31	2 Palpable nodes
32	4 Palpable nodes
33	6 Palpable nodes
…continues until…	
39	18+ Palpable nodes
**PRINCIPAL GROWTH STAGE 4: BOOTING**	
40	Flag leaf visible but still rolled
41	Flag leaf is fully expanded
43	Inflorescence occupies 25% of flag leaf sheath
45	Inflorescence occupies 50% of flag leaf sheath
47	Inflorescence occupies 75% of flag leaf sheath
49	Inflorescence fills flag leaf sheath but no florets are exposed
**PRINCIPAL GROWTH STAGE 5: INFLORESCENCE EMERGENCE**	
51	First florets just visible through flag leaf collar
53	Inflorescence upper branches exposure
56	Inflorescence lower branches exposure
59	Inflorescence fully exposed and peduncle exposure
**PRINCIPAL GROWTH STAGE 6: FLOWERING**	
60	Sporadic open florets
*61*	10% of florets open
62	20% of florets open
63	30% of florets open
… continues until…	
69	90 to 100% of florets open
**PRINCIPAL GROWTH STAGE 7: SEED DEVELOPMENT**^‡^	
71	Watery ripe
73	Early milk
75	Medium milk
77	Late milk
**PRINCIPAL GROWTH STAGE 8: RIPENING**^‡^	
81	Early dough
83	Soft dough
85	Hard dough
87	Fully ripe
89	Over-ripe
**PRINCIPAL GROWTH STAGE 9: SENESCENCE**	
90	Partial leaf yellowing
91	Stems 10% senesced
92	Stems 20% senesced
93	Stems 30% senesced
…continues until…	
99	Stems 90 to 100% senesced

*The two digit code consists of a combination of the principal growth stage code (tens place) and secondary stage code (ones place)*.

**Figure 1:**
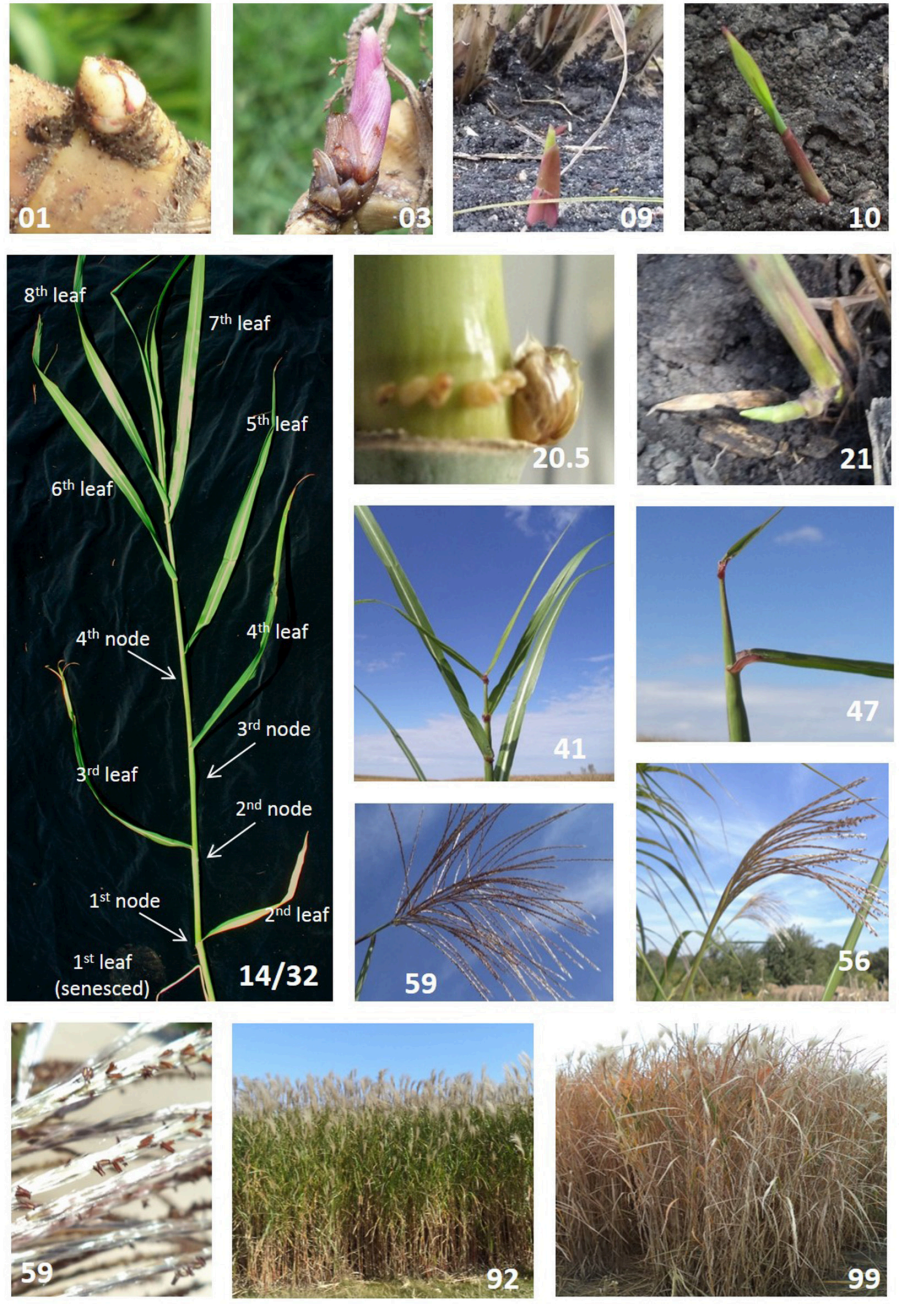
Select *Miscanthus* × *giganteus* morphological development stages as described using the BBCH scale (Table 2). Each picture highlights a single developmental stage: 01 beginning of bud swelling; 03 end of bud swelling; 09 emergence through soil surface; 10 first visible leaf lamina; 14/32 stem with eight fully expanded leaves (14) and 4 nodes (32); 20.5 swollen axillary bud (~8–10 mm); 21 one tiller on main shoot; 41 flag leaf is fully expanded; 47 inflorescence occupies 75% of flag leaf sheath; 56 inflorescence lower branches exposed; 59 inflorescence fully exposed and peduncle exposure; 59 showing anther details; 92 stems 20% senesced; 99 stems 90–100% senesced.

*See Figure 1 for corresponding images. Stages are assessed on individual stems*.

‡ *Stages never or rarely observed in commercial M. × giganteus clones. Based on Meier, (2001) general grass descriptions and adapted to perennial grasses using by Sanderson et al. (1997)*.

### BBCH scale description and codification for *M. × giganteus*

#### Principal growth stage 0: bud development

Stage 0 describes plant development beginning with rhizome buds. It is also applicable to axillary buds growing from aerial stems, which have also been proposed as propagules (Boersma and Heaton, 2014), as in sugarcane. Rhizome bud development goes from beginning of bud swelling (stage 01) through emergence when leaves break through the soil surface (stage 09). Bud swelling ends (stage 03, Figure 1) as buds break (stage 05), the first true leaves elongate past protective bud scales (Figure 1), and leaf laminae grow toward the soil surface (Table 2). In the establishment year, bud development typically begins in late spring, depending on planting date and weather conditions. In older stands, stem emergence typically begins when soil temperatures are consistently above 10°C or cumulative degree days above 0°C are higher than 650 (Hastings et al., 2009).

#### Principal growth stage 1: leaf development

This principal stage is based on the total number of leaves present on the stem and does not differentiate between green or senesced leaves. Observers can choose to assess only green leaves or include senesced leaves, depending on their goals. The proportion of senesced leaves present at a given point is presented in stage 9, senescence. New leaves are not counted until fully expanded as indicated by a visible ligule. New leaves continue to emerge until the stem fully flowers and senesces, or is winter-killed. *M*. × *giganteus* typically has less than 20 leaves on a stem; therefore, we suggest here that secondary stages advance by every two leaves (Table 2). For example, a stem with two fully expanded leaves would be at growth stage 11, where the principal growth stage 1 (leaf development) is given in the tens place and the secondary growth stage is given in the ones place as 2 leaves/2 = 1. Similarly, a stem with 8 leaves would be at growth stage 14 (Figure 1). Should a stem have 15 leaves, a mesostage can be added using a decimal point, and the stem would be at growth stage 17.5. Although uncommon, especially if only following green leaves, stems that produce more than 19 leaves would be staged as 19.5, the maximal value for this growth stage.

#### Principal growth stage 2: tillering

Because this scale is applied to individual stems coming from a belowground rhizome network, tillering in this sense refers to new stems developed from axillary buds on the monitored stem. *M*. × *giganteus* stems are unbranched and rarely produce tillers, however, some swollen axillary buds could appear on the base of the stem, especially if the stem is damaged (e.g., from hail or herbivory). Rarely, these buds will produce new stems and it is even more seldom that these stems will reach the canopy level. Secondary stages in this case correspond to the number of tillers produced per stem. The norm is no tillers on a stem (stage: 20) though infrequently one or two tillers may be present (stage 21 or 22).

Presence of swollen buds could be recorded within this principal growth stage since it could have impacts on phenology and plant physiology. These stages would be included as decimal points; 20.1: presence of partially swollen axillary bud (~2 mm), 20.5: axillary bud fully swollen (~8–1.0 mm, Figure 1), 20.9: Bud breaking. Finally, in the case of tillering, the scale continues to be applied to the main stem (Table 2).

#### Principal growth stage 3: stem elongation

In *M*. × *giganteus*, stem elongation occurs soon after emergence and continues until the culm flowers or is winter-killed. This stage is of prime importance since it tracks the development of the principal harvestable structure. Stem elongation is addressed by counting the number of aboveground palpable nodes present on the stem, that is, the number of nodes that can be felt by gently pressing along the stem. We suggest that, as in leaves, secondary stages progress every two nodes (Table 2). For example, a stem with eight fully expanded leaves and four palpable nodes would be coded as 14/32 (Figure 1), indicating 4 ^*^ 2 = 8 leaves in principal growth stage 1 (leaf development) and 2 ^*^ 2 = 4 palpable nodes in principal growth stage 3 (stem elongation). If more detail is needed, odd number of nodes could be coded as mesostages using the tenths place. For example, if a stem has seven nodes it should be staged as 33.5, indicating 3 ^*^ 2 = 6 nodes and 0.5 indicating the presence of another node (giving a total of seven nodes). As in leaf development, stems with more than 19 nodes (biologically rare, Uwatoko et al., 2016) will all be staged as 39.5.

#### Principal growth stage 4: booting

This principal stage refers to the progression of the inflorescence through the appearance of the flag leaf sheath. It starts when flag leaf is visible but lamina still rolled (stage 40). Stage 41 occurs when the flag leaf is fully expanded (Figure 1), indicating the end of leaf development. Booting ends when the inflorescence fills the entire flag leaf sheath and no florets are yet exposed (stage 49). Intermediate secondary stages distinguish the proportion of the sheath occupied by the inflorescence. Since this could be hard to identify and track we recommend using generic landmarks and assigning them in 25% increments. In consequence, only three secondary growth stages are required to allocate these morphological changes evenly: 43, 45, and 47 (Table 2, Figure 1). For instance, stage 43 occurs when the inflorescence fills 25% of the sheath, stage 45 when the inflorescence occupies 50% and so on. First florets may not appear through the collar of the flag leaf and may grow instead through the sheath. In this case the stem will still be staged as 49.

#### Principal growth stage 5: inflorescence emergence

The *M*. × *giganteus* inflorescence is a panicle of racemose branches with paired and pedicellate spikelets (Hodkinson and Renvoize, 2001). This stage starts with upper spikelets coming out of the flag leaf collar (stage 51) and ends once the panicle is fully exposed and the peduncle is visible (stage 59). In general terms, secondary stages could be characterized by: upper branch exposure (borne on the upper half of the main axis, stage 53), lower branch exposure (borne on the lower half of the main axis lower branches, stage 56, Figure 1) and peduncle exposure (stage 59, Table 2, Figure 1). If more detail is required, secondary growth stages could be expressed as the exposed percentage of the panicle. This requires a trained observer to estimate the relative proportion of a partially exposed panicle. Note that stage 59 may not occur in environments where the growing season is not long enough or hard frost events are frequent in middle to late fall. In this case inflorescence emergence may stop before the panicle is fully exposed. Exposed florets may continue to open.

#### Principal growth stage 6: flowering

This stage is defined as the proportion of exposed florets with emerged anthers. It starts with sporadic exposed anthers (stage 60) until full flowering (stage 69) when anthers are present throughout the entire inflorescence; secondary stages are attributed in 10 percent increments (e.g., stage 65: 50% of anthers are exposed, Table 2, Figure 1). Flowering proceeds basipetally through the inflorescence. Anther exposure can occur while the inflorescence is still emerging and, consequently, the inflorescence could fully flower even though the inflorescence is not fully exposed.

*M*. × *giganteus* is a sterile clone and no seed is produced so flowering represents the last reproductive stage. In order to expand the scope of this scale to other varieties and *Miscanthus* spp., grain maturity stages were included. These stages were based on general grass descriptions from Meier (2001) as adapted to perennial grasses (e.g., *P. virgatum*) by Sanderson et al. (1997).

#### Principal growth stage 7: seed development

At the beginning of seed development (stage 71) the total number of cells in the endosperm is established and grains have a watery ripe consistency; first grains may have reached half their final size. Gradually, seeds increase solid (e.g., starch, protein) concentrations and reach their final size at medium milk stage (75). Seed development ends at late milk stage (77).

#### Principal growth stage 8: ripening

Most of the seed dry weight is accumulated during the ripening stage. Along with the continued increment in starch and protein concentration, water content decreases, increasing grain hardness. Secondary stages are characterized by pressing the grain with a fingernail. Stage 83 is when grain content is soft and the fingernail impression is not held; 85 is when a fingernail impression remains after the test. Stage 87 represents when the grain is hard and difficult to break with a thumbnail and finally stage 89 is when the grain cannot be dented.

#### Principal growth stage 9: senescence

This stage describes the senescence progression of the stem during a growing season and it is based on leaf senescence without differentiating between possible causes, e.g., stress or seasonal cues. In order to provide a better approach for comparative purposes it should be reported as the proportion of the total number of leaves present at any given point. A leaf is consider senesced once 50% or more of the laminae has senesced. For simplicity, it could also be quantified as a visual estimate of the senesced proportion of the entire plant. For instance, a plant with five senesced leaves out of a total of 25 could be considered 20% senesced and coded as 92 (Figure 1). Stages 91–99 represent the progressive senescence of leaves on the stem, from 10 to 90%, respectively, at 10% increments (Table 2, Figure 1). This stage could start very early in the growing season once the stand reaches canopy closure and low light quality triggers senescence in lower canopy leaves.

### Using morphological data to assess phenology

#### Estimating growth stage progression

**Figure 2:**
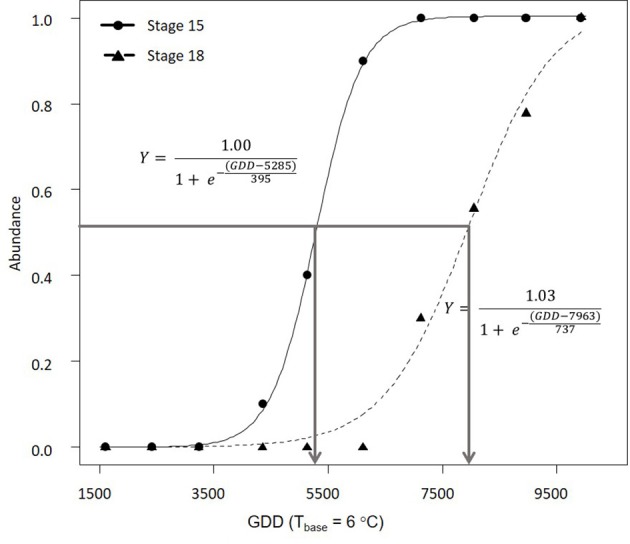
Progression of 2-year-old *Miscanthus* × *giganteus* through BBCH developmental stages 15 and 18 in 2016. The horizontal line indicates 50% of stems at certain stage or higher and the arrows indicate the estimated onset date for each stage. Logistic equation for each progression is also presented.

In 2016, morphological development was observed biweekly in 2-year-old *M*. × *giganteus*. Data were used to illustrate the steps required to estimate progression of morphological development stages using the cumulative abundance of a given stage (Table 3). For example on July 11 there were four stems at stage 15 or higher (2 at 15 and 2 at 15.5) and 0 stems at stage 18. By September 6 all sampled stems were at stage 15 or higher, and seven were at stage 18 or higher (4 at 18, 2 at 18.5 and 1 at 19). Note that abundance is expressed as a proportion and thus is not dependent on equal sample sizes between dates. Graphing cumulative abundance of growth stages 15 and 18 over time produced sigmoid curves that were used to derive the onset date of each stage (Figure 2). The onset date of stage 15 was July 13 (5282 GDD) and the onset date of stage 18 was August 19 (7963 GDD).

**Table 3:** Raw explanatory data showing number of stems of *Miscanthus* × *giganteus* BBCH stage 15 or higher as measured during the 2016 growing season in Sorenson farm, Iowa.

**BBCH stage**	**Jun-29**	**Jul-11**	**Jul-25**	**Aug-9**	**Aug-23**	**Sep-6**	**Sep-21**
	**Number of stems at a given stage**						
15	0	2	0	0	0	0	0
15.5	1	2	0	0	1	0	0
16	0	0	6	0	0	0	0
16.5	0	0	1	2	0	1	0
17	0	0	2	4	2	1	0
17.5	0	0	0	1	1	0	0
18	0	0	0	3	3	4	0
18.5	0	0	0	0	1	2	4
19	0	0	0	0	0	1	3
19.5	0	0	0	0	1	0	1
	**Cumulative sum of stems at a given stage or higher**						
15	1	4	9	10	9	9	8
18	0	0	0	3	5	7	8
	**Cumulative proportion of stem**						
15	0.1	0.4	0.9	1	1	1	1
18	0	0	0	0.2	0.56	0.78	1

*Sample size was 10 stems for all sampling dates but for 23-Aug, 6-Sept and 21-Sep with 9, 9, and 8 stems, respectively*.

**Figure 3:**
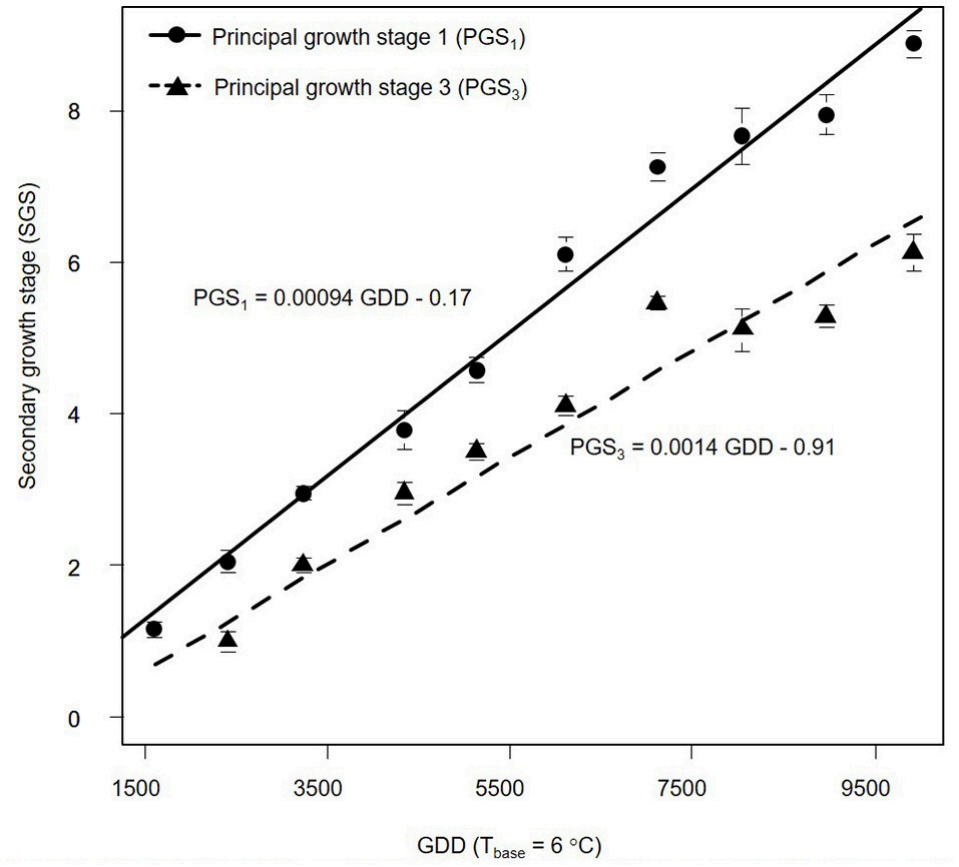
Progression of *Miscanthus* × *giganteus* principal growth stage 1 (leaf development) and 3 (stem elongation) over growing degree days (GDD). Stages were based on the BBCH scale developmental scale. Each observation is the average of 10 stems. Vertical lines represent ± 1 standard error; see text for details on the summary statistic methods. Linear equations for each principal growth stage are provided and principal growth stage developmental rate corresponds to the slope of each equation.

We also estimated PGSDR for principal growth stage 1 (leaf development; PGSDR_1_) and 3 (stem elongation; PGSDR_3_) as the slope of the linear regression between average principal growth stage per sampling date and thermal time in GDD (Figure 3). For example, PGSDR_1_ = 0.00094 means that almost a thousandth of a secondary growth stage is developed per GDD. Its inverse (1053) represents the number of GDD required to complete a secondary growth stage. Given that secondary growth stages advance every two leaves, leaf appearance rate is double PGSDR_1_ (0.00188 leaf GDD^−1^) and the phyllochron is half of PGSDR_1_ (526.5 GDD leaf ^−1^).

#### Growth stage and summary statistics

The *M*. × *giganteus* morphological development data collected over the 2016 growing season was also used to demonstrate the calculation of the average and standard deviation of principal growth stages 1 and 3 (Table 4).

**Table 4:** Explanatory data showing the principal growth stages (1 = leaf development and 3 = stem elongation) and secondary growth stages of five *Miscanthus* × *giganteus* stems over four sampling dates in 2016 in Sorenson farm, Iowa.

**Principal Growth Stage**	**May-19**	**Jun-13**	**Jul-11**	**Sep-6**
**1: LEAF DEVELOPMENT**				
	11.5	12.5	14	18.5
	11.5	12.5	15.5	18
	11	13.5	15	18.5
	11.5	13	15.5	16.5
	11	13	14.5	18
*BBCH*_1_	$\frac{56.5}{5}$ = 11.3	$\frac{64.5}{5}$ = 12.9	$\frac{74.5}{5}$ = 14.9	$\frac{89.5}{5}$ = 17.9
_*S*_*BBCH*_1___	$\sqrt{\frac{0.3}{5}}$ = 0.24	$\sqrt{\frac{0.7}{5}}$ = 0.37	$\sqrt{\frac{1.7}{5}}$ = 0.58	$\sqrt{\frac{2.7}{5}}$ = 0.74
**3: STEM ELONGATION**				
	30	32	33.5	36
	30	31.5	34	35.5
	30	31.5	34	35.5
	30	32	33.5	35
	30	32.5	33.5	35.5
*BBCH*_3_	$\frac{150}{5}$ = 30	$\frac{159.5}{5}$ = 31.9	$\frac{168.5}{5}$ = 33.7	$\frac{177.5}{5}$ = 35.5
_*S*_*BBCH*_3___	$\sqrt{\frac{0}{5}}$ = 0	$\sqrt{\frac{0.7}{5}}$ = 0.37	$\sqrt{\frac{0.3}{5}}$ = 0.24	$\sqrt{\frac{0.5}{5}}$ = 0.31
*BBCH*_*complete*_	11.3	12.9/31.9	14.9/33.7	17.9/35.5

*Average stage, standard deviation at principal growth stage i (BBCH_i_), and the complete growth stage for each sampling date are presented*.

## Discussion

We used peer-reviewed literature and field observations to modify the BBCH morphological development scale (Lancashire et al., 1991) for *M*. × *giganteus* and related species. The inclusion of principal growth stages related to seed development, absent today in commercial clones, allows easy adaption and application of the scale to other species including new seeded hybrids with higher adaptability and stress tolerance (Clifton-Brown et al., 2017). The detailed, yet flexible scale framework should enable inter-comparison of *M*. × *giganteus* studies, and facilitate phenological research and crop management.

### Comparison with other scales

The simplicity of the *Miscanthus* BBCH scale permits conversion of previous phenological descriptions into a standardized form regardless of the specific research topic, allowing comparison of results from multiple study-specific scales. For example, the five-point scale (1 = 80–100% green, 2 = 60–80% green, 3 = 40–60% green, 4 = 20–40% green, 5 = <20% green) used by Purdy et al. (2015) to characterize *Miscanthus* spp. seasonal carbohydrate dynamics corresponds to stages 90 through 99 in our BBCH scale. Similarly, in the scale Fonteyne et al. (2016) used (0 = no flowering, 1 = flag leaf formed, 2 = panicle emergence, 3 = anthesis, 4 = end of anthesis) to study rhizome and shoot frost tolerance, stages 1, 2, 3, and 4 correspond to BBCH stages 41, 59, principal stage 6, and 69, respectively. However, Fonteyne's (2016) stage 3 (anthesis) does not provide enough detail about anthesis progression through the panicle to allow for a secondary BBCH stage to be attributed. Additionally, “no-flowering” (Fonteyne's stage 0) is not specific enough to link to any BBCH stage.

While the above scales were used to assess plant physiology with implications for plant breeding, phenology is also widely used in ecosystem modeling assessments. The phenological description used by Miguez et al. (2009) to parameterize the eco-physiological model WIMOVAC for *M*. × *giganteus* is different than the one used in MISCANFOR (Hastings et al., 2009), however, they could both be coded consistently using the BBCH scale reported here. Likewise, the scale Hastings et al. (2009) used to describe *M*. × *giganteus* growth and development counting GDD using a base temperature of above 0°C (GDD_0_) could be expressed using our BBCH code system in the following ways: shoot emergence (BBCH stage 09) starts at GDD_0_ > 650, mean air temperature > 10°C, and when the photoperiod is longer than 12 h. Leaf development (BBCH stage 10) starts at GDD_0_ > 850 and ends (BBCH stage 19) when leaf area index < 8, 3 days below 10°C, 3 days below the wilting point, or GDD_0_ = 2,200. Finally, plant senescence starts (BBCH stage 90) when there are 6 days below 10°C, one frost day, or 30 wilting days. Similarly, phenology assessment can also be expressed using our BBCH scale in the Agricultural Production Systems Simulator (APSIM) re-parametrization for *M*. × *giganteus* developed by Ojeda et al. (2017) based on Trybula et al. (2015). They used GDD = 1,000 from emergence to stem elongation. This equates to stage 09 through principal growth stage 3 in our scale. Similarly, GDD = 800 from stem elongation to flowering, is from principal growth stage 3 to principal growth stage 5, and GDD = 300 from flowering to full senescence, corresponds to principal growth stage 5 to stage 99 in our scale.

### Scale uses and applications

In addition to interoperability, another important advantage of our BBCH scale is its scalability, made possible by the arithmetically meaningful nature of its numerical indices and coding. Scalability allows measurement on a practical scale, i.e., on individual stems, rather than depending on sampling non-discreet plants or undertaking the sampling of an entire population. The method presented here to estimate summary statistics from a multiple-stem sample has been modified from Kalu and Fick (1981) and Moore et al. (1991) to incorporate multiple development states of *M*. × *giganteus* in parallel. This is a crucial attribute of our coding system because not only do individual stems experience multiple stages in parallel notably leaf development (1) and stem elongation (3), but it is a virtual certainty that multiple stages would be observed among stems at a field scale, and not considering parallel stages could produce misleading estimates. Take, for example, a sample of stems where one has only four leaves (stage 12) and the other already two palpable nodes (stage 31). The calculated mean growth stage for the sample would be 22, indicating the average stem has two tillers, which is completely erroneous, and biologically unlikely since tillering in *M*. × *giganteus* is rare. In contrast, estimating summary statistics per principal growth stage and presenting them together separated by oblique strokes provides a more accurate description.

Repeated observations reveal progression of morphological stages and enable calculation of important *M*. × *giganteus* phenology parameters. For example, onset dates are useful to coordinate agronomic practices and help characterize the effect of environmental and management factors on the growth cycle. We presented a regression-based method to model phenological progression over time estimating onset and duration of stages. This method, adapted from Schirone et al. (1991), is flexible enough to use with other nonlinear models. For example, an asymptotic exponential curve could be used if the initial lag phase is very short; see Archontoulis and Miguez (2013) for a complete review of other possible nonlinear models. Another convenient feature is that it allows use of different thresholds to define onset dates. However, sigmoidal curve interpolation errors are larger at the beginning and end of the curve so, for a better estimation of onset dates, thresholds should be within the linear section of the curve (Cornelius et al., 2011). Moreover, this method is not restricted to calendar date as a measure of time, but can be used for estimations using accumulated thermal time or light interception to further parameterize ecosystem models. Principal growth stage developmental rate also describes progression of morphological stages. While calculation of onset dates provides a better description of the progression of an individual secondary growth stage, PGSDR estimates the rate of progression of the entire principal growth stage and requires fewer observations to be estimated. If PGSDR is constant for all secondary growth stages, the difference between onset dates of two sequential stages is equivalent to PGSDR.

In conclusion, the proposed extended BBCH scale provides a detailed and accurate description of *M*. × *giganteus* morphological stages, using a simple and intuitive two-digit code method. Overall, the proposed modified BBCH scale will enhance effective communication by presenting a precise and uniform framework of terminology and quantitative metrics that enable analytical assessment. The scale takes into account special features of *M*. × *giganteus* such as, its perennial life cycle, lack of seed production, rhizomatous growing habit and indiscreet plants. Moreover, it easily accommodates the development of the large number of leaves and nodes produced by *M*. × *giganteus* stems. Used together with methods presented here to spatially upscale and assess phenology, this coding system provides support to the entire *M*. × *giganteus* community by enabling intercomparison across scientific studies and providing growers with developmentally appropriate crop management guidance.

## Author contributions

MT collected all data required for the research, developed the current scale and was the main author of the paper. EH supervised MT and provided substantial contributions to the conception and writing of the paper.

### Conflict of interest statement

The authors declare that the research was conducted in the absence of any commercial or financial relationships that could be construed as a potential conflict of interest.
